# Detection of atrial fibrillation in persons aged 65 years and above using a mobile electrocardiogram device

**DOI:** 10.1007/s12471-023-01828-6

**Published:** 2023-11-28

**Authors:** Fenna Daniëls, Tanwier T. T. K. Ramdjan, Balázs Mánfai, Ahmet Adiyaman, Jaap Jan J. Smit, Peter Paul H. M. Delnoy, Arif Elvan

**Affiliations:** Department of Cardiology, Isala Heart Centre, Zwolle, The Netherlands

**Keywords:** Atrial fibrillation, Screening, Primary care, Mobile electrocardiogram device, Stroke prevention

## Abstract

**Background:**

Untreated atrial fibrillation (AF) often results in increased morbidity and mortality. Opportunistic AF screening in persons aged ≥ 65 years is recommended to identify patients with AF in order to prevent AF-related complications.

**Objective:**

The aim of this study was to assess the feasibility of screening persons for AF with the Kardia mobile electrocardiogram device (MED) and to determine the percentage of newly detected AF cases by selective population screening in the Netherlands.

**Methods:**

Persons aged ≥ 65 years, without a medical history of AF, in nursing homes, at public events or visiting the general practitioner (GP) were approached to participate. A Kardia MED smartphone ECG (sECG) was recorded and the CHA_2_DS_2_-VASc score was calculated. An automated AF algorithm classified the sECGs as ‘sinus rhythm’, ‘AF’ or ‘unclassified’. In the case of AF, participants were referred to their GP. All sECGs were assessed by blinded experts.

**Results:**

A total of 2168 participants were screened for AF. According to the expert’s interpretation, 2.5% had newly detected AF, of whom 76.4% never experienced palpitations and 89.1% had a CHA_2_DS_2_-VASc score ≥ 2. The algorithm result was unclassified in 12.2% of cases, of which 95.5% were interpretable by experts. With expert opinion as the gold standard and excluding unclassified sECGs, the Kardia MED’s negative and positive predictive value for detecting AF was 99.8% and 60.0%, respectively.

**Conclusion:**

Screening for AF using the Kardia MED is feasible and results in 2.5% newly detected AF cases. Expert interpretation of algorithm outcomes AF and unclassified is recommended.

## What’s new?

- The AliveCor Kardia device is an appropriate atrial fibrillation (AF) screening tool in primary care, although algorithm-derived outcomes AF and unclassified need to be reviewed by experts.
- Screening for AF in persons aged ≥ 65 years results in 2.5% newly detected AF cases.
- The Kardia device’s negative predictive value for detecting AF is 99.2%, whereas the positive predictive value is 60.0%

## Introduction

Atrial fibrillation (AF) is the most common cardiac arrhythmia with a lifetime risk of 37%, and its complications such as heart failure and stroke result in increased morbidity and mortality [1]. Adequate anticoagulant treatment can reduce the 5% average annual stroke risk by approximately 60% [2]. However, approximately 40% of AF patients are asymptomatic, resulting in underdiagnosis and undertreatment [3]. Earlier studies showed that 27–45% of patients diagnosed with stroke and AF were not aware of their existing AF [4, 5]. Therefore, early detection and treatment of AF is of great importance.

Opportunistic screening has shown promising results in detecting AF and cost-effectiveness by preventing strokes, resulting in a class IB indication in the 2020 ESC guidelines for opportunistic screening in patients aged ≥ 65 years [6–9]. Mobile electrocardiogram (ECG) devices (MEDs) with AF assessment algorithms have been introduced as a participant-friendly screening device. The Kardia MED (KardiaMobile; AliveCor Inc, Mountain View, CA, USA) previously showed sensitivity and specificity for detecting AF of 54.4–98% and 97–99.4%, respectively [10–12]. However, the varying results require that more and larger comparative studies be performed, including studies that determine the feasibility of the device’s implementation in healthcare systems.

The aim of this study is to examine the feasibility of screening persons ≥ 65 years old for AF, including those with newly detected AF, and to validate the Kardia MED as a screening instrument for application in transmural care.

## Methods

The Kardia device was distributed in ten general practices in the region of Zwolle, the Netherlands, where participants were included in the study by the general practitioner (GP) or practice nurse. Moreover, a screening team was present during influenza vaccination, at a trade fair for household products (*Huishoudbeurs*, Home Fair, Amsterdam) and visited nursing homes. Persons aged ≥ 65 years were asked to participate and informed consent was obtained. Exclusion criteria were a history of AF and a pacemaker or an implantable cardioverter defibrillator. A single smartphone ECG (sECG) was recorded, the CHA_2_DS_2_-VASc score was calculated and participants were asked if they experienced palpitations [13]. Participants with palpitations were those who had not visited their GP with palpitations or in whom AF had not been diagnosed. Afterwards, a physician assessed all sECGs, blinded for the algorithm classification. In the case of inconsistencies two experts independently reassessed the sECG. sECGs were interpreted as ‘sinus rhythm (SR)’, ‘AF’ or ‘uninterpretable’. In the case of AF, participants were referred to their GP for further evaluation.

### Kardia MED

Once the Kardia application has been downloaded and installed on a smartphone, recording can commence. The Kardia device uses ultrasound to create a 30‑s ECG trace. With the device placed next to the smartphone, two fingers of the left hand are placed on the left electrode and two fingers of the right hand on the right electrode. An AF algorithm is implemented, based on the criteria R‑R interval irregularity and the absence of P‑waves. The algorithm classifies the recording as ‘possible AF’, ‘SR’, ‘unclassified’ and ‘bradycardia’ (heart rate < 50 bpm) or ‘tachycardia’ (heart rate ≥ 100 bpm). For data analysis, bradycardia and tachycardia were considered to be unclassified. In tachycardic participants, the algorithm could indicate possible AF.

#### *Data analy*sis

Statistical analysis was performed using IBM SPSS Statistics, version 27.0 (IBM Corp., Armonk, NY, USA). The physician’s sECG interpretation was considered the gold standard. Continuous variables were presented as mean ± standard deviation, categorical variables as frequencies. To compare groups, Pearson’s chi-squared test or Fisher’s exact test were used for dichotomous variables. Differences in means of continuous data were tested by performing Student’s *t*-test (two groups) or one-way ANOVA (> 2 groups) or, in the case of non-normally distributed data, by performing the Mann-Whitney U test (two groups) or Kruskal-Wallis test (> 2 groups). Univariate and multivariate logistic regression were used to define predictors for AF.

## Results

**Fig. 1:**
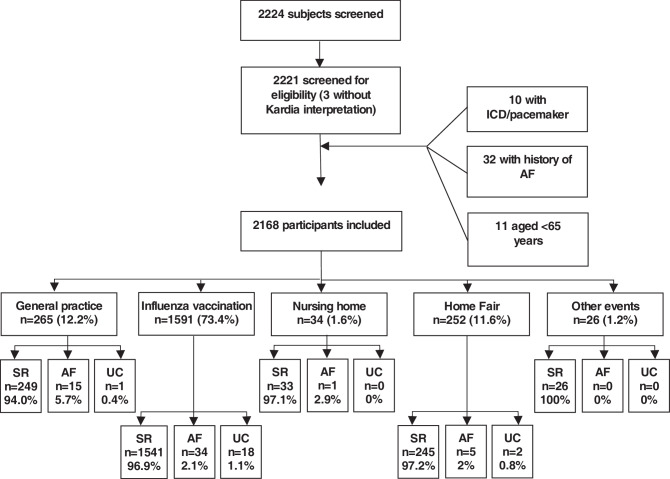
Study flow chart.* ICD* implantable cardioverter defibrillator, *AF* atrial fibrillation, *SR* sinus rhythm, *UC* unclassified

A total of 2224 participants were included between June 2018 and November 2019. Of these, 56 participants did not meet the inclusion criteria and were excluded from analysis, resulting in 2168 participants with a mean age of 73 ± 6 years; 57.9% were female and 88.8% of the participants had a CHA_2_DS_2_-VASc score ≥ 2. All characteristics of the participants are shown in Tab. 1. Of the participants, 73.4% were included during their influenza vaccination and 12.2% during regular visits to their GP. The study flow chart is shown in Fig. 1.

**Table 1:** Participant characteristics per rhythm group

	SR (*n* = 2092)	AF (*n* = 55)	UN (*n* = 21)	*p*-value	SR (*n* = 1840)	AF (*n* = 65)	UN (*n* = 263)	*p*-value
	*Expert opinion*				*Algorithm outcome*			
Age, years	73 ± 6	77 ± 6	77 ± 8	< 0.001	73 ± 6	76 ± 6	74 ± 6	< 0.001
Female gender	1222 (58.8%)	23 (41.8%)	10 (47.6%)	0.025	1095 (60.0%)	28 (43.1%)	132 (50.2%)	< 0.001
Heart failure	129 (6.2%)	6 (10.9%)	2 (9.5%)	0.317	107 (5.9%)	6 (9.2%)	24 (9.1%)	0.086
Hypertension	988 (47.7%)	28 (50.9%)	12 (57.1%)	0.622	868 (47.7%)	40 (61.5%)	120 (45.6%)	0.067
Stroke/TIA/thrombo-embolism	217 (10.5%)	9 (16.4%)	2 (9.5%)	0.368	192 (10.5%)	10 (15.4%)	26 (9.9%)	0.423
Vascular disease	296 (14.3%)	8 (14.5%)	9 (42.9%)	0.001	259 (14.2%)	14 (21.5%)	40 (15.3%)	0.243
Diabetes mellitus	288 (13.9%)	8 (14.5%)	1 (4.8%)	0.475	238 (13.1%)	11 (16.9%)	48 (18.3%)	0.062
Palpitations	496 (23.7%)	13 (23.6%)	4 (19.0%)	0.882	441 (24.0%)	17 (26.2%)	55 (20.9%)	0.492

Values are mean ± standard deviation or numbers with percentages

*SR* sinus rhythm, *AF* atrial fibrillation, *UN* unclassified, *TIA* transient ischaemic attack

According to the algorithm, the AF prevalence was 3.0%, whereas experts found a prevalence of 2.5%. The algorithm showed 12.2% unclassified outcomes; experts were able to interpret 95.5% of these sECGs. Due to poor trace quality, 1.0% of all sECGs were uninterpretable. Of the unclassified outcomes, 4.6% were interpreted by experts as AF. Including the unclassified/uninterpretable outcomes in the no AF cases, the algorithm showed a sensitivity and specificity for detecting AF of 70.9% and 98.8%, respectively. Negative (NPV) and positive predictive values (PPV) were 99.2% and 60.0%, respectively. Excluding the unclassified outcomes resulted in a sensitivity and specificity of 90.7% and 98.6%, respectively, with a NPV of 99.8% and PPV of 60.0%. All outcomes are shown in Tab. 2.

**Table 2:** Algorithm outcomes and expert conclusion

		Expert conclusion			
		Sinus rhythm	Atrial fibrillation	Uninterpretable	Total
*Algorithm outcome*	Sinus rhythm	1832	4	4	1840
	Atrial fibrillation	21	39	5	65
	Unclassified	239	12	12	263
*Total*		2092	55	21	2168

### False-negative or false-positive results

**Fig. 2:**
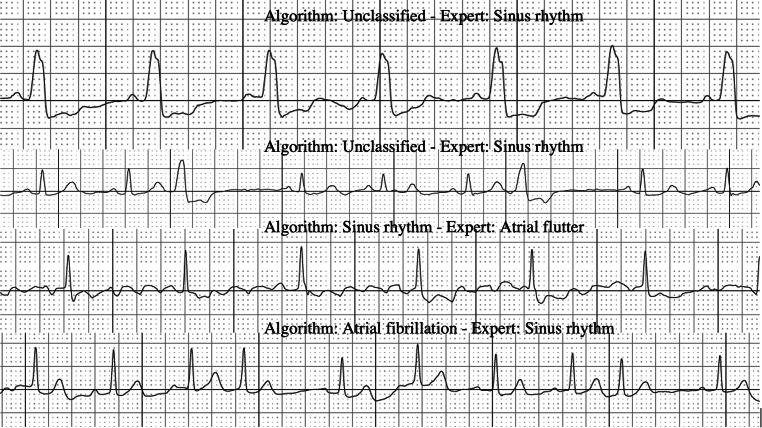
Examples of differences in algorithm classification and expert interpretation

Of the 4 false-negative results, 2 showed atrial flutter and 2 had normal AF. The 21 false-positive results consisted of sECGs with premature ventricular or atrial complexes or broad QRS complexes. Examples are shown in Fig. 2.

### Atrial fibrillation

Of the participants diagnosed with AF, 23.6% experienced palpitations. Their mean age was higher than that of the SR group: 77 ± 6 years (*p* < 0.001); 41.8% were female and 89.1% had a CHA_2_DS_2_-VASc score ≥ 2.

There was no significant difference in palpitations between the SR and AF group (*p* = 0.990). Of all the components of the CHA_2_DS_2_-VASc score, only gender (*p* = 0.012) and age (*p* < 0.001) differed significantly between SR and AF. Age and male gender were independent predictors for detecting AF with an odds ratio of 1.095 (95% confidence interval (CI) 1.049 ± 1.142) and 2.008 (95% CI 1.156 ± 3.489), respectively (Tab. 3).

**Table 3:** Univariate and multivariate logistic regression analysis to predict atrial fibrillation detection by experts

Predictors	OR	95% CI	*p*-value	OR	95% CI	*p*-value
	*Univariate analysis*			*Multivariate analysis*		
Age per year	1.095	1.051 ± 1.141	< 0.001	1.095	1.049 ± 1.142	< 0.001
Male gender	1.977	1.149 ± 3.402	0.019	2.008	1.156 ± 3.489	0.013
Heart failure	1.829	0.769 ± 4.349	0.172			
Hypertension	1.132	0.663 ± 1.935	0.649			
Stroke/TIA/thrombo-embolism	1.677	0.810 ± 3.473	0.164			
Vascular disease	0.999	0.467 ± 2.135	0.998			
Diabetes mellitus	1.058	0.495 ± 2.263	0.884			
Palpitations	0.999	0.532 ± 1.875	0.996			

*OR* odds ratio, *CI* confidence interval, *TIA* transient ischaemic attack

The prevalence of AF differed among screening locations; in comparison to other screening locations, the GP detected significantly more cases of AF (5.7%, *p* = 0.001). Excluding the 10% participants with previous thromboembolic events resulted in 2.4% AF cases. Screening during influenza vaccination resulted in 2.1% of newly detected AF cases. Of the participants in the Home Fair group, 34.1% experienced palpitations, which was significantly more compared with other locations (*p* = 0.002).

### CHA_2_DS_2_-VASc score

The CHA_2_DS_2_-VASc score was significantly higher in the nursing home group compared with the other locations (*p* < 0.001), with GP second highest. Only incidence of heart failure and gender did not occur significantly more often in the nursing home compared with the other locations. Excluding the nursing home group, participants visiting the GP had hypertension (*p* < 0.001) and diabetes mellitus (*p* = 0.004) significantly more often than those in other locations.

## Discussion

This study shows that AF screening in a high-risk population using the Kardia device is feasible, although expert interpretation of the 15.1% unclassified and AF outcomes is required. The Kardia algorithm’s performance in our study is influenced by unclassified outcomes and supported by the literature. Lau et al. showed the Kardia device’s sensitivity and specificity for detecting AF to be 87% and 97%, respectively, with 12-lead ECG interpretation as the gold standard [10]. Desteghe et al. compared the Kardia algorithm with a 12-lead ECG in 265 patients. The algorithm’s sensitivity and specificity were 54.5% and 97.5%, respectively, with a NPV of 96% and PPV of 66.7% [12]. Unfortunately, neither study reported the percentage of unclassified outcomes, impeding a reliable comparison of sensitivity with that in our study. Other authors reported NPVs of 98.4% in two studies comparing the algorithm with a 12-lead ECG, and 98% when comparing the algorithm with expert interpretation [14–16].

Although the NPV of the Kardia device is high in our study, enabling sufficiently reliable SR classification, the 35.0% false-positive outcomes are a limitation. This finding is in accordance with the previously mentioned studies, with PPVs ranging between 54.8% and 80% [12, 15, 16]. This might have been caused by premature complexes, as confirmed by the Hartwacht study, where experts interpreted 8% of the cases with an algorithm outcome of AF as SR with ectopic beats [16].

Not only the cases classified by the algorithm as AF should be reassessed. The majority of the unclassified sECGs could be interpreted by experts and therefore should always be reviewed. High proportions of unclassified sECGs and the expert’s ability to assess these sECGs were reported previously. The Hartwacht team showed 17% unclassified algorithm outcomes and 8% uninterpretable expert interpretations [16]. Other studies reported even higher percentages of unclassified outcomes: 19.5–27.5% [15, 17].

This study showed an overall prevalence of newly detected AF of 2.5%, with a higher prevalence at the GP. Although we assumed this difference could have been caused by selection bias from possibly screening symptomatic participants, the proportion of participants experiencing palpitations was not higher than in the other groups. Another explanation could be that most participants were included during cardiovascular risk management consultations, including patients at higher risk of AF. This is also supported by the higher incidence of hypertension and diabetes mellitus compared with other screening locations besides the nursing home. The relatively low rate of AF cases in nursing homes could have been a result of a higher prevalence of already detected AF.

Several screening studies have been performed with varying AF prevalences. In 2007, Fitzmaurice et al. studied 14,802 participants aged ≥ 65 years, randomised to (1) 12-lead ECG single screening, (2) 12-lead ECG single screening in the case of an irregular pulse, or (3) no screening. Detection rates of new AF cases were 1.62%, 1.64% and 1.04%, respectively [18]. The STROKESTOP study in 2015 included 7173 participants aged 75–76 years who recorded a sECG twice a day for 2 weeks using the Zenicor device (Zenicor Medical Systems, Stockholm, Sweden), resulting in an AF prevalence of 3.0% [19]. The REHEARSE-AF study in 2017 included 1001 patients aged ≥ 65 years who were randomised to a 12-month screening programme using the Kardia device twice a week or routine care and resulted in 3.8% and 1.0% AF, respectively [20]. Both the STROKESTOP and the REHEARSE-AF study showed the diagnostic benefits of periodic screening. However, the appropriate and cost-effective screening frequency remains to be defined [9]. The study by Kaasenbrood et al., resembling part of our study with AF screening during seasonal influenza vaccination using the MyDiagnostick device (MyDiagnostick Medical, Maastricht, The Netherlands) revealed 1.1% newly detected AF. Although all age groups were screened, no new cases were detected in participants aged below 60 years, which could be an explanation for the higher percentage of AF cases in our study [7].

A recently published EHRA position paper on searching for AF underlines the current challenges in AF detection and suggests the use of clinical risk scores (MR-DASH or C_2_HEST) to better refine target populations [21, 22]. Additionally, a monitoring time of 2 weeks or longer is preferred to maximise the possibility of identifying subjects with AF [23].

### Limitations

One major limitation of this study is that we did not collect data on the use of anticoagulation or an extensive medical history. Participants could not adequately recall their medication and in the setting of this population screening programme we did not include a search in medical records. Moreover, it remains unclear whether implementation of anticoagulation after opportunistic screening provides similar protection against stroke to when AF is diagnosed clinically.

Secondly, expert interpretation was considered the gold standard, which could still be wrongly interpreted. However, this is in line with the ESC guidelines, with a class 1B recommendation that definite diagnosis of AF in screen-positive cases is established after the physician reviews the sECG recording of ≥ 30 s [9].

Moreover, to facilitate rapid screening we only asked if participants experienced palpitations before screening for AF symptoms. Although AF can account for a broad spectrum of symptoms, we considered palpitations to be the most common symptom. Additionally, there is a wide variety in the number of participants included at different screening locations, requiring caution in the interpretation of differences in participant characteristics and screening outcomes. The yield of AF detection depends on the a priori chance of having AF, which is higher in our population of persons aged 65 years and above compared with the general population. A selection bias, selecting patients with higher comorbidity, could not be excluded. Therefore, extrapolation to the general population should be done carefully.

In our study, no cost-effectiveness analysis was performed. The previously mentioned influenza vaccination screening study showed that screening in primary care during seasonal influenza vaccination in a population aged ≥ 65 years would have an estimated probability of 99.8% for being cost-effective at a conservative willingness to pay of € 20,000/QALY [6]. The cost-effectiveness of screening is also implied by the STROKESTOP study [19].

### Recommendation

In addition to the benefit of AF screening as described in the ESC guidelines, screening with the Kardia device is feasible when reviewing unclassified and AF outcomes [9]. Although the cost-effectiveness needs to be examined, our recommendation would be to implement screening at vaccination programmes. Ideally, persons at higher risk of new AF should be included and could be identified using risk scores [21, 22]. Preferably, persons with a high CHA_2_DS_2_-VASc score, thus requiring initiation of oral anticoagulation, should be selected. Persons already on oral anticoagulants might be excluded due to the lack of clinical consequences. Future research should examine the implementation of repeated or intensified screening programmes.

When persons are informed prior to the screening event, 2–3 min per person is required. It is recommended that cardiologists and GPs cooperate closely to facilitate quick reviewing: 15.1% of sECGs in the current study.

## Conclusion

Our study confirms the feasibility of using the Kardia device as an appropriate AF screening tool in primary care to provide contemporary AF treatment and prevent AF-related complications, given its high NPV and substantial diagnostic yield in a high-risk population. However, algorithm-derived outcomes AF and unclassified need to be reviewed by experts.
